# Can an average of thresholds at 2 kHz and 4 kHz substitute for the threshold at 3 kHz in pure tone audiometry? A study based on the Korean National Health and Nutrition Examination Survey 2010–2012

**DOI:** 10.1371/journal.pone.0201867

**Published:** 2018-08-07

**Authors:** Ju Yeon Kim, Sung Wan Byun, Seung-Ho Shin, Mi Sun Chun

**Affiliations:** Department of Otorhinolaryngology, College of Medicine, Ewha Womans University, Seoul, Republic of Korea; Center for Healthy Start Initiative, NIGERIA

## Abstract

**Background:**

When evaluating hearing disability in medicolegal cases, an average of thresholds at several frequencies is calculated using pure tone audiometry. Occasionally, there are instances in which thresholds at certain frequencies are omitted. One typical example is the threshold at 3 kHz (H3k). The American Academy of Otolaryngology–Head and Neck Surgery Committee on Hearing and Equilibrium (1995) suggested that the average of thresholds at 2 kHz and 4 kHz (H24k) could replace H3k for a comparison of results between studies. However, to the best of our knowledge, there is no report in the literature that compares H3k and H24k.

**Objective:**

This study aimed to investigate the agreement between H3k and H24k.

**Methods:**

This study is based on the Korea National Health and Nutrition Examination Survey (KNHANES) 2010–2012, which was conducted by the Korean government. A total of 18,472 participants (unweighted) who represented 39,357,497 Koreans (weighted) were included. To verify the agreement of H3k and H24k, a paired t-test, Cohen’s d, Pearson’s correlation, Cronbach’s coefficient, intraclass correlation coefficient (ICC), a Bland–Altman plot, and linear regression analysis were used.

**Results:**

The means of H3k and H24k were 16.2 dBHL and 16.6 dBHL, respectively. They were significantly different in a paired t-test (p<0.0001), which resulted from the large sample size. In contrast, the effect size (Cohen’s d) was 0.02, which meant that the two groups nearly overlapped. The means showed strong correlation: Pearson’s correlation coefficient = 0.92, Cronbach’s alpha = 0.96, and ICC = 0.92. A strong linear predictive relationship between H3k and H24k was found: *y* = –0.6821 + 1.0186*x*, where *x* = H24k, *y* = H3k, and p<0.0001. However, the Bland–Altman plot showed large upper and lower limits of agreement (LOA) of 15.0 dBHL and –15.8 dBHL, respectively. Irrespective of age and degree of the four-tone average (0.5, 1, 2, and 3 kHz) hearing loss or thresholds at 2 kHz, 3 kHz, and 4 kHz, the absolute LOAs were greater than 10 dBHL.

**Conclusions:**

Despite a very strong correlation between the two thresholds, H3k and H24k showed clinically large LOAs. Therefore, it would be improper to substitute H24k for H3k in an individual requesting a hearing disability rating. However, since the overall means of the H3k and H24k samples were nearly equal, H24k can replace H3k for a mean comparison of results between studies. This result supports the 1995 Committee on Hearing and Equilibrium guideline.

## Introduction

When a hearing disability rating is requested because of an accident or work-related injury, pre-accident hearing threshold should be reviewed to calculate apportionment, which is the allocation of causation among multiple factors that caused or significantly contributed to the injury and the resulting impairment. Depending on the type of accident, a two, three, four, or six-tone average can be used for the rating. However, occasionally there are instances in which thresholds at certain frequencies have not been tested before accidents occur. Typically, the absent frequency is 3 kHz because it is not routine to test the interoctave hearing threshold at H3k in many hearing centers. The American Academy of Otolaryngology–Head and Neck Surgery Committee on Hearing and Equilibrium (1995) suggested that H3k could be replaced with the average of thresholds at 2 kHz and 4 kHz (H24k) to compare results between retrospective studies when H3k was unavailable [1]. However, to the best of our knowledge, no report has verified the relationship between these two thresholds.

The Korea National Health and Nutrition Examination Survey (KNHANES) 2010–2012 was a complex, stratified, multistage, probability-cluster survey of a representative sample of the non-institutionalized civilian population in South Korea. The survey provided reliable audiological data for stratified samples representing the entire Korean population. This study aimed to investigate the agreement between H3k and H24k using data from KNHANES.

## Methods

### Study design, population, and data collection

The KNHANES study design, population, and data collection were conducted as described in our previous study [2]. A total of 23,621 individuals (10,611 males and 13,010 females), representing 47,761,044 individuals (23,884,864 males and 23,876,180 females) in South Korea participated in the KNHANES survey from 2010 to 2012. Pure tone audiometry data were acquired from 8,102 males, representing 19,772,135 Korean men, and 10,370 females, representing 19,585,362 Korean women. The participants were between the ages of 12 and 82 years as the sample size of people over the age of 82 was too small for valid analyses. The mean age of the study participants was 46.49 years (unweighted) and 41.61 years (weighted) (Table 1). Therefore, audiometric data for 36,944 ears, which represented 78,714,994 ears, were analyzed.

**Table 1:** Demographic data of participants in this study (subset of Korea National Health and Nutrition Examination Survey [KNHANES]).

Period of Survey	2010–2012	
**Type of study sampling design**	Simple random sampling design	Complex survey design
**Subjects**	18,472 participants	39,357,497 (representing population)
**Male**	8,102 (43.86%)	19,772,135 (50.24%)
**Female**	10,370 (56.14%)	19,585,362 (49.76%)
**Mean age (years)**	46.49	41.61
**Age range (years)**	12–82	12–82

### Audiometric measures

The audiometric measures were conducted in the same way as described in our previous study [2]. It can be briefly described as follows. For pure-tone audiometric testing, a Entomed SA-203 audiometer (Entomed; Lena Nodin, Sweden) was used. Only air conduction thresholds were measured with supra-auricular headphones in a sound-treated booth. Automated testing was programmed according to a modified Hughson-Westlake procedure with appropriate masking.

### Ethics statement

The KNHANES obtained written informed consent from each participant prior to the survey. The Institutional Review Board of Ewha Womans University Mokdong Hospital approved this research (IRB No. EUMC 2015-10-056).

### Statistical analysis

Statistical analyses and graphing were performed using R software (version 3.2.1; The R Foundation, Vienna, Austria, URL http://www.R-project.org).

## Results

**Fig 1:**
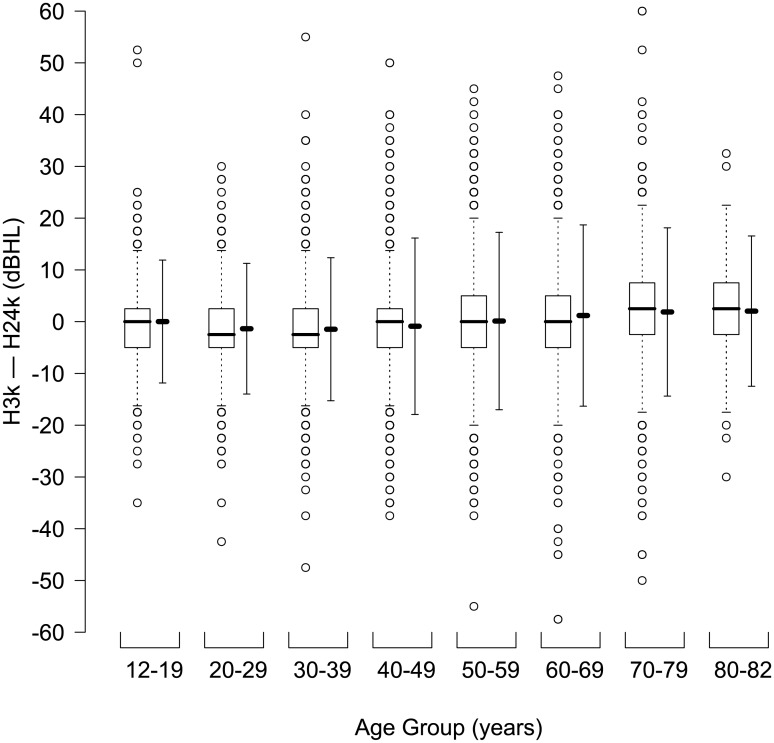
Box-and-whisker plot (left) combined with mean difference and limit of agreement (LOA, right) for the decibel difference between the threshold at 3 kHz (H3k) and the average of thresholds at 2 kHz and 4 kHz (H24k) according to eight age groups (weighted N = 39,357,497 × 2 ears, age: 12–82 year-old). The box height is the interquartile range (IQR = Q3 –Q1) of the decibel difference for each age. The thick horizontal line in the box is the median (Q2). Outliers are marked as empty circles below the vertical dotted line from the box (Q1–1.5 IQR) and above the dotted line (Q3 + 1.5 IQR). This boxplot shows that the median and the interquartile range of difference between H3k and H24k are distributed around zero, irrespective of age group. Therefore, it can be interpreted that H3k and H24k had very similar mean values in all cases. In addition, the absolute limit of agreement in all age group was very large irrespective of age. dBHL: decibel hearing level; H2k: threshold at 2 kHz; H3k: threshold at 3 kHz; H4k: threshold at 4 kHz; IQR: interquartile range; Q1: first quartile (25 percentile); Q2: second quartile (median, 50 percentile); Q3: third quartile (75 percentile).

**Fig 2:**
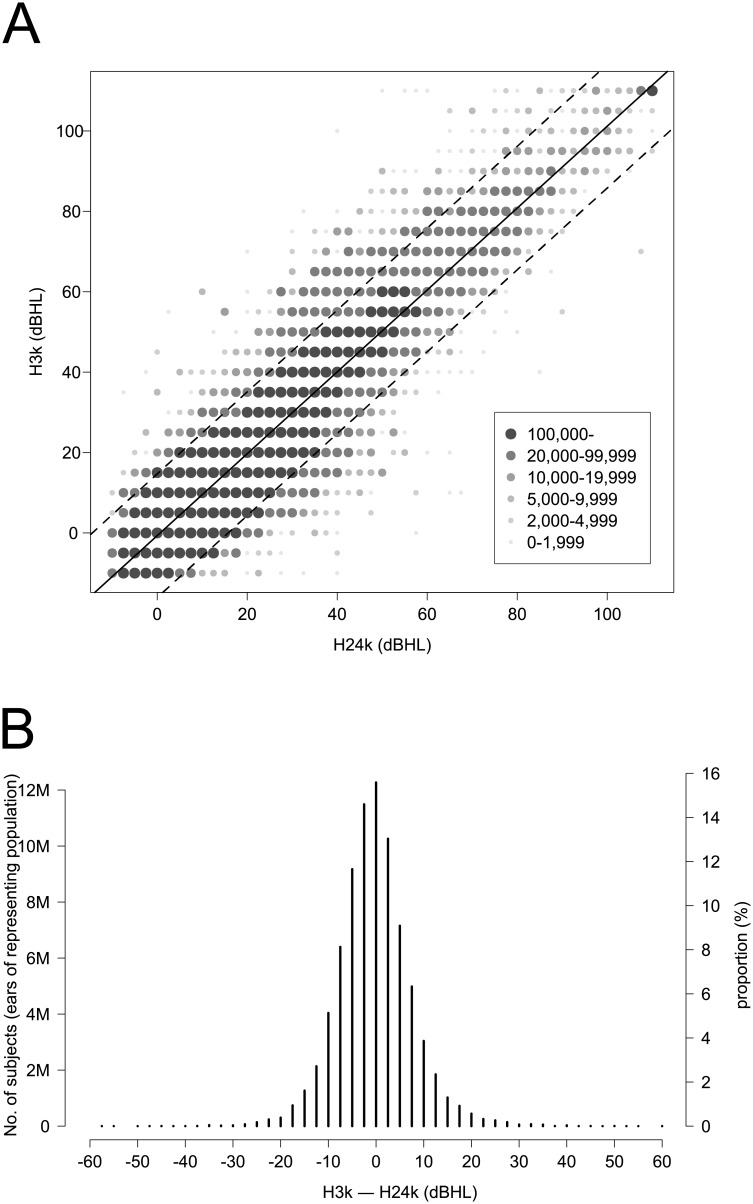
(A) Scatter plot between the threshold at 3 kHz (H3k) and the average of thresholds at 2 kHz and 4 kHz (H24k). (B) Distribution of the difference between H3k and H24k (weighted N = 39,357,497 × 2 ears). The scatter plot shows a statistically strong correlation between H3k and H24k (R = 0.92, p<0.0001). The predictive linear regression equation was *y* = –0.6821 + 1.0186*x* (*x* = H24k, *y* = H3k, p<0.0001, R^2^ = 0.849). The difference between the H3k and H24k displays a distribution pattern that is similar to a normal distribution. M: million; dBHL: decibel hearing level.

**Fig 3:**
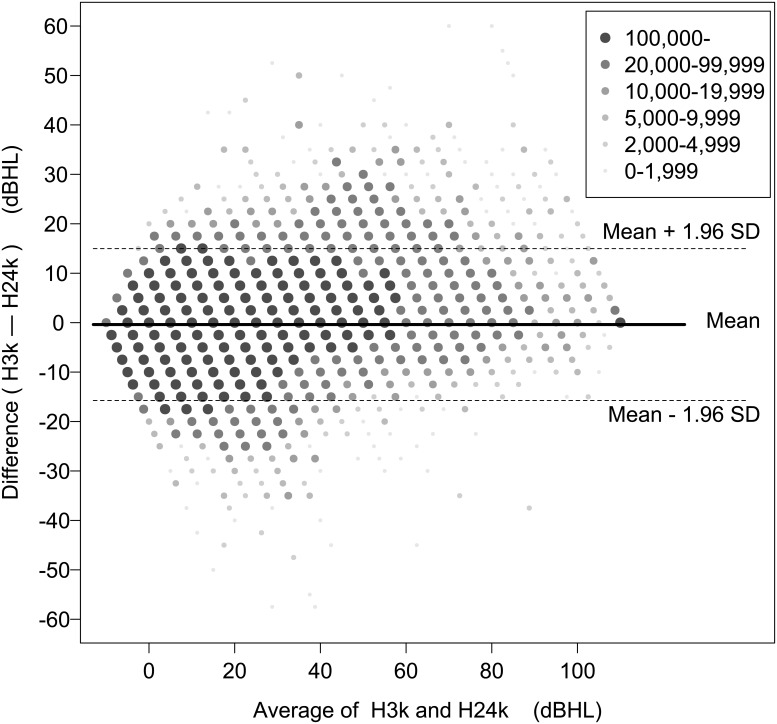
Bland–Altman plot for the threshold at 3 kHz (H3k) and the average of thresholds at 2 kHz and 4 kHz (H24k) (weighted N = 39,357,497 × 2 ears). This plot shows that the absolute limit of agreement is large, which means that H24k cannot be a substitute for H3k. Thick line (center): mean difference (–0.38); dashed lines (top and bottom): upper and lower limits of agreement (15.0 and –15.8, respectively); dBHL: decibel hearing level; SD: standard deviation.

**Fig 4:**
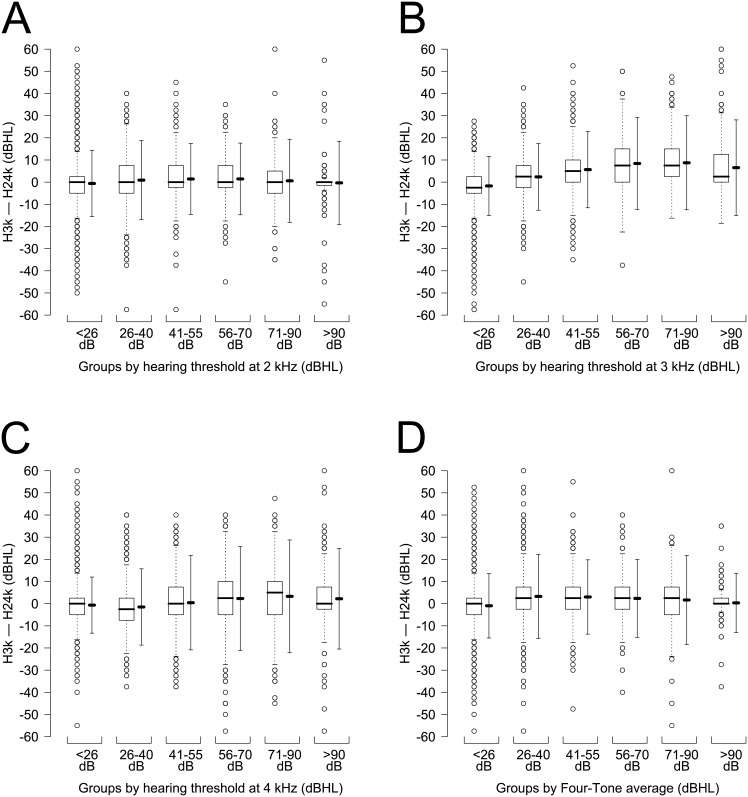
Box-and-whisker plot (left) for the mean difference and limit of agreement (right) of difference between the threshold at 3 kHz (H3k) and the average of thresholds at 2 kHz and 4 kHz (H24k) against the threshold groups. (A) 2 kHz; (B) 3 kHz; (C) 4 kHz; and (D) four-tone (0.5, 1, 2, and 3 kHz) average (weighted N = 39,357,497 × 2 ears). These graphs show that the degree of hearing thresholds at the three frequencies and at the four-tone average did not affect the distribution of the difference between H3k and H24k. However, the upper and lower limits of agreement are roughly greater than 10 dBHL and less than –10 dBHL, respectively. ※Threshold groups are divided as follows: normal (<26 dBHL), mild (26–40 dBHL), moderate (41–55 dBHL), moderately severe (56–70 dBHL), severe (71–90 dBHL), and profound (>90 dBHL); dBHL: decibel hearing level.

In this study, the difference was obtained when H24k was subtracted from H3k. To compare H24k and H3k, a paired t-test was performed and an effect size (Cohen’s d) was calculated. To show the distribution of the difference by age in a complex random sampling design considering sampling weights, a box-and-whisker plot, which included the mean difference and the limit of agreement (LOA), was made (Fig 1). To find the correlation and agreement between H3k and H24k, Pearson’s correlation coefficient and Cronbach’s alpha were calculated. A scatter plot was made to determine the characteristics of correlation between H3k and H24k (Fig 2A); the mean difference between H3k and H24k is shown in Fig 2B. To evaluate the agreement between H3k and H24k, a Bland–Altman plot was made (Fig 3) in which the upper and lower LOA were calculated. The mean difference and the LOA were used to determine whether or not the difference was affected by the degree of threshold at 2 kHz, 3 kHz, and 4 kHz, and at a four-tone average (0.5, 1, 2 and 3 kHz) (Fig 4). A p <0.05 was considered to be statistically significant.

The means of H3k and H24k were 16.2 dBHL and 16.6 dBHL, respectively. Though the difference (–0.38 dB) was very small, H3k and H24k were significantly different in a paired t-test (p<0.0001), which probably resulted from the very large sample size. The effect size (Cohen’s d) was 0.02, which meant that the two groups nearly overlapped. Fig 1 depicts the age-related medians and the interquartile ranges of difference, which was around zero within narrow ranges; note that the *y*-axis is the difference (H3k –H24k). This indicates that H3k and H24k had very similar mean values for all ages. In addition, the absolute LOA in each age group was very large—roughly > 10 dB.

Pearson’s correlation coefficient was 0.92 (p<0.0001), which indicated that the two thresholds showed a strong positive correlation, while the difference showed a normal distribution pattern (Fig 2A and 2B). The predictive linear regression equation for Fig 2A was determined to be *y* = –0.6821 + 1.0186*x*, where *x* = H24k, *y* = H3k, p<0.0001, and R^2^ = 0.849, which was strongly significant. Cronbach’s alpha was 0.96, which indicated that the internal consistency between the two thresholds was excellent. The intraclass correlation coefficient (ICC) with 0.92 showed strong resemblances and consistency between the H3k and H24k variables (Table 2) [3]. While the mean difference of –0.38 dB was very small, the maximum and minimum differences were 60.0 dB and –57.5 dB, respectively, which were large. As shown in Fig 3, the upper and lower LOAs of the Bland–Altman plot were 15.0 dBHL and –15.8 dBHL, respectively, which were clinically large and indicate that it was not possible to substitute H24k for H3k.

**Table 2:** Correlation analyses between the threshold at 3 kHz (H3k) and the average of thresholds at 2 kHz and 4 kHz (H24k).

	H3k—H24k
**Pearson’s r**	0.92 (p<0.0001)
**Cronbach’s alpha**	0.96
**ICC**	0.92 (p<0.0001)

ICC (1,1): intraclass correlation, one-way random, single measures

Fig 4 shows that the hearing thresholds at 2 kHz, 3 kHz, and 4 kHz, and at the four-tone average were divided into six standard audiometric categories: normal (<26 dBHL), mild (26–40 dBHL), moderate (41–55 dBHL), moderately severe (56–70 dBHL), severe (71–90 dBHL), and profound (>90 dBHL). The degree of the thresholds and the four-tone average (0.5, 1, 2, and 3 kHz) did not affect the difference. However, the upper and lower LOAs were greater than 10 dBHL and less than –10 dBHL, respectively, in all cases, which meant that they were clinically large.

## Discussion

Our research question was to determine whether or not H24k could be substituted for H3k in pure tone audiometry. To determine if H24k can be a substitute for H3k, the agreement of the two factors should be evaluated. Many methods have been suggested for the evaluation of agreement, although no single estimate can provide a complete picture about data reliability [4]. First, we performed a paired t-test to determine the difference between H24k and H3k. Though the mean difference was –0.38 dB, which was clinically tiny, the means of the two thresholds were statistically different. A possible explanation for this result is that as the sample size increases, the statistical power also increases such that the smallest effect size can be detected; in this scenario, a tiny effect can be found to be statistically significant. This can happen unless the effect size is zero. To verify the effect size, Cohen’s d was calculated and determined to be 0.02. This confirmed that the mean difference was small, which indicated that the means of H3k and H24k were almost the same. Therefore, the means of all the samples of the two thresholds were statistically similar, which meant that H24k could replace H3k for a comparison of the results between studies.

To evaluate the relationship between H3k and H24k, various analyses, including Pearson’s correlation, Cronbach’s alpha, and intraclass correlation, were conducted. The analyses showed statistically strong correlations.

However, the Bland–Altman plot (Fig 3) showed that the LOA was too large for the average to represent the threshold at the individual level [5]. Study participants were divided into various groups by age, and the degree of hearing loss at several thresholds and the four-tone average was used to determine whether these variables affected the LOA. We found that these groupings did not affect the large LOA. By examining the specific data, the proportion of H3k that was equal to H24k, or was located between the thresholds at 2 kHz and 4 kHz, was merely 51.3% of all cases. Thus, we interpreted these results to indicate that H24k cannot replace H3k in terms of being usable data for an individual requesting a hearing disability rating.

We believe that the lack of agreement between individual H3k and H24k can be explained using the following reasoning. Various pathologic processes can influence hair cells, stria vascularis, neurons, and the central auditory nervous system, and can damage diverse areas of the auditory system in different combinations. In a pure tone audiogram, the hearing threshold at each frequency is the result of the function of the inner and outer hair cells, stria vascularis, and neurons working together based on the histopathologic evidences and cytocochleogram matched by the audiogram [6, 7]. According to tonotopic organization, the hair cells that correspond to a specific frequency are located from the apical turn to the basal turn. Therefore, the hair cells that are relevant to hearing at 3 kHz are located among the cells related to hearing at 2 kHz and 4 kHz. Those cells can be damaged by various insults; these insults may influence the cells at the wide region of the cochlea, but sometimes affect only specific or multiple discrete regions. This explains why H24k is not always similar to H3k in each individual. The difference between H3k and H24k shows a normal distribution in Fig 2 because of the various ways in which insults randomly affect the cochlea in each individual. However, since the distance among the hair cells at those frequencies is very small, there may be a tendency for the mean of H3k to be remarkably similar to the mean of H24k in the overall population.

## Conclusions

While a very strong correlation was found between the two thresholds of H24k and H3k, there was also a large LOA. Therefore, it would be improper to substitute H24k for H3k for an individual requesting a hearing disability rating. We recommend that the H3k at the individual client level should be routinely assessed in cases where information at the frequency could be important diagnostically or medicolegally. In contrast, the overall means of the samples of the two thresholds were statistically similar, which indicates that H24k can replace H3k for a comparison of results between studies. This result supports the 1995 Committee on Hearing and Equilibrium guideline.

## Supporting information

S1 Table Detailed numeric data in this study. (CSV)
